# Distinguishing Neurocognitive Processes Reflected by P600 Effects: Evidence from ERPs and Neural Oscillations

**DOI:** 10.1371/journal.pone.0096840

**Published:** 2014-05-20

**Authors:** Stefanie Regel, Lars Meyer, Thomas C. Gunter

**Affiliations:** Department of Neuropsychology, Max Planck Institute for Human Cognitive and Brain Sciences, Leipzig, Saxony, Germany; Charité University Medicine Berlin, Germany

## Abstract

Research on language comprehension using event-related potentials (ERPs) reported distinct ERP components reliably related to the processing of semantic (N400) and syntactic information (P600). Recent ERP studies have challenged this well-defined distinction by showing P600 effects for semantic and pragmatic anomalies. So far, it is still unresolved whether the P600 reflects specific or rather common processes. The present study addresses this question by investigating ERPs in response to a syntactic and pragmatic (irony) manipulation, as well as a combined syntactic and pragmatic manipulation. For the syntactic condition, a morphosyntactic violation was applied, whereas for the pragmatic condition, such as “*That is rich*”, either an ironic or literal interpretation was achieved, depending on the prior context. The ERPs at the critical word showed a LAN-P600 pattern for syntactically incorrect sentences relative to correct ones. For ironic compared to literal sentences, ERPs showed a P200 effect followed by a P600 component. In comparison of the syntax-related P600 to the irony-related P600, distributional differences were found. Moreover, for the P600 time window (i.e., 500–900 ms), different changes in theta power between the syntax and pragmatics effects were found, suggesting that different patterns of neural activity contributed to each respective effect. Thus, both late positivities seem to be differently sensitive to these two types of linguistic information, and might reflect distinct neurocognitive processes, such as reanalysis of the sentence structure versus pragmatic reanalysis.

## Introduction

Event-related potentials (ERPs) are well suited to depict the timing of language comprehension, as well as to dissociate the neurocognitive processes engaged in the comprehension of linguistic and non-linguistic information. Research on language comprehension has shown distinct ERP components related to the processing of semantic/pragmatic (N400) and syntactic information (P600). Because an N400 response, that is, a centroparietal negativity between 300–500 ms after stimulus onset, reliably emerged for different types of semantic manipulations, this component seems to be sensitive to lexical processing and might reflect access to meaning (for review see [1], [2]). The N400 component is typically affected by semantic expectancy [3],[4] and congruity of a word on both sentence and discourse levels (for review see [5]).

For manipulations of syntactic information, however, a P600 component is most robustly obtained (for review see e.g., [6]). This positivity emerges around 500 ms after stimulus presentation and displays a centroparietal scalp distribution. The P600 has been reported first for syntactically anomalous sentences, such as “*The broker persuaded to sell the stock”, in which the verb-argument structure was violated [7]. Besides, the P600 appears to be sensitive to a variety of further syntactic violations, such as violations of phrase structure [8], [9], subcategorization [10], and morphosyntactic constraints [11]–[13]. A P600 response has also been obtained for syntactically complex or ambiguous sentences [14]–[16], suggesting that this ERP component is sensitive to different syntactic subprocesses. According to Friederici [17], the P600 is a reflection of structural repair and reanalysis processes. Alternatively, the P600 is considered as a common indicator of syntactic operations [6], [15]. Since these early reports of syntax-related positivities, the language-specificity of the P600 has been controversially debated. Findings of an influence of probability of stimulus occurrence, as well as of salience of violation type on the P600 amplitude, led to alternative interpretations. In a study by Coulson et al. [18], both ungrammatical and improbable stimuli elicited comparable late positivity effects with a similar scalp distribution, suggesting that the P600 may rather be an instantiation of the domain-general P300 component. An influence of stimulus probability and sentence complexity on the amplitude of P600 was also shown in a study by Gunter et al. [13]. While the observed modulations of the P600 by non-linguistic manipulations are in favor of a domain-general, P300-related interpretation, there is also some evidence against this view [19], [20]. Recent findings showing a sensitivity of the P600 component to semantic information further raise the question whether this component reflects specific or rather common processing mechanisms. A P600 response was seen for syntactically well-formed sentences that were semantically manipulated in various ways (for review see [21]). The amplitude of P600 was modulated by semantic expectancy [22], thematic role and semantic reversal anomalies [23]–[27], as well as by semantic incongruencies on the sentence and discourse levels [28]–[32]. Moreover, for both syntactically and semantically well-formed, but pragmatically/conceptually more complex sentences, P600 effects reliably emerged, such as for figures of speech [33]–[36], jokes [37], in response to sentence verification tasks [38], and discourse comprehension [39]. Whenever there is a strong semantic relation between the elements of a sentence, only a P600 response, but no additional N400 effect was obtained for semantic-thematic anomalies [24], [25], [27], [40]. However, similar results were also found in cases when the elements of a sentence were not strongly semantically related [33], [34], [41]. To date, there is an ongoing debate about the functional significance of these so-called ‘semantic P600’ effects. On the one hand, findings of P600 for thematic role anomalies have been associated with structural processes engaged in revision of the actor and undergoer role assignments (e.g., [41], [42]). On the other hand, P600 effects in response to semantic reversal anomalies have been interpreted as monitoring effects, resulting from a conflict between an algorithmic and a heuristic processing stream [27], [43]. In a recent review on ‘semantic P600’ effects, these ERP effects are attributed to an update of the current mental representation by incoming information [21].

The observation of P600 effects for various types of syntactic as well as semantic/pragmatic manipulations gives rise to the question of whether this component reflects common neurocognitive processes, or rather functionally distinct processing mechanisms related to the processing of particular information types. In the current study, the functional characteristics of the P600 component are examined by comparison of P600 responses to a syntactic anomaly, as well as to a pragmatic ambiguity (i.e., irony). The applied experimental paradigm crosses both types of manipulations, thereby allowing for a direct comparison of ERP responses for the combined condition with those of the single syntactic and pragmatic condition.

In addition to the ERP analysis, the oscillatory neural activity is analyzed by time-frequency analysis (TFA) in order to capture potential processing aspects that are not visible using ERPs. TFA allows an investigation of the synchronization and desynchronization of neural populations resulting from the (de)coupling of functionally related assemblies of neurons (e.g., [44]). In response to an incoming event, functionally related neural assemblies can be assumed to fire synchronously in a given frequency band, leading to frequency-specific changes (i.e., increase or decrease of power) of the oscillatory neural activity measurable at the scalp surface. With regard to language comprehension, previous EEG studies employing TFA often reported changes in the alpha band (8–12 Hz) for lexical retrieval [45], syntactic anomalies (e.g., [46]), and pragmatic ambiguities [36], but also for storage of linguistic information in verbal working memory [47]. On the role of synchronization/desynchronization of alpha power, however, there is still an ongoing controversy (for review see e.g., [48]). According to current accounts, alpha synchronization might reflect inhibitory processes, whereas alpha desynchronization seems to be associated with an inhibition release [48]. Besides changes in the alpha band, synchronization in the theta band (4–7 Hz) has been observed for semantic [49], [50], and pragmatic information processing [36], as well as for syntactic anomalies [46], among other linguistic manipulations. These findings suggest a common role of theta activity for language processing.

Based on the findings of the afore-mentioned studies, the following predictions can be made. A syntax-related P600 component is predicted for the syntactic anomaly (i.e., a morphosyntactic violation) in relation to syntactically correct sentences as observed in previous studies (e.g., [13], [18], [51]). Additionally, a left anterior negativity (LAN) between 300–500 ms after stimulus onset is predicted for syntactically anomalous sentences. A LAN response has often been reported for subject-verb disagreements [22], [52], and seems to exhibit some sensitivity to morphosyntactic information processing, presumably reflecting syntactic analysis processes (for review see [53]). The observation of a biphasic ERP pattern consisting of LAN-P600 for the syntactic anomaly might index syntactic information processing. Moreover, for the pragmatic manipulation, a replication of the P600 component for ironic compared to literal sentences (i.e., an irony-related P600) is expected as seen recently [33], [34], [36]. Further, a P200 component emerging around 200 ms post-stimulus onset related to irony is predicted as shown previously [34].

The following patterns of results are hypothesized for the P600 effect. If independent neural networks contribute to the emergence of both the syntax-related and the irony-related P600, the neural activity reflected in the P600 amplitude for the combined condition (i.e., syntactically anomalous irony) can be expected to sum up, resulting in an additive effect (see e.g., [54]). Similarly, the observation of differences in the scalp distribution between the syntax-related and the irony-related P600 would imply independent patterns of neural activity contributing to both effects, and point towards distinct neurocognitive processes reflected by both potentials (e.g., syntactic and pragmatic information processing). Moreover, the TFA of the respective P600 time window should reveal differences in the oscillatory neural activity, particularly in the theta band, between the syntax and the pragmatics effect. Differences in the brain oscillations in combination with the expected ERP results would support an engagement of different neural assemblies in the processing of syntax and pragmatics. If, in contrast, neither distributional differences between the syntax-related and irony-related P600, nor an additive effect on the P600 amplitude for the combined condition is observed, this finding would indicate that both the syntax-related and the irony-related P600 effects are elicited by similar neural networks, and are not independent of each other. Additionally, the TFA should reveal no differences in the oscillatory neural activity in the P600 time window for the processing of syntactic and pragmatic information. Such a pattern of results would imply an involvement of similar neural assemblies in the processing of both types of linguistic information, substantiating the assumptions of common neurocognitive processes reflected by the P600 [18], [27].

## Materials and Methods

### Participants

Forty students (20 female, mean age 25.1 years (standard deviation (SD) 2.19)) from the University of Leipzig participated in the experiment and were paid for their participation. All were right-handed, native speakers of German, with normal or corrected-to-normal vision, and no attested language impairment. This study was approved by the ethics committee of the University of Leipzig. All participants gave informed written consent before taking part in the study. The study was conducted in accordance with the ethical principles stated in the Declaration of Helsinki.

### Stimulus Material and Procedure

As stimulus material, a set of 120 German target sentences preceded by three to four context sentences was presented. Depending on the contextual information, for which a pleasant or disappointing event was described, target sentences achieved either a literal or ironic meaning. Thereby, irony conveyed a different meaning of what had been stated literally. For each target sentence, two types of contexts were created (promoting respective literal or ironic sentence interpretations), thus resulting in a total of 240 stories (i.e., combinations of context sentences and target sentence). For the pragmatic condition, the target sentences achieved an ironic interpretation with respect to the previous context, causing a pragmatic ambiguity (for examples, see Table S1). For the correct condition (i.e., literal correct sentences), and the pragmatic condition (i.e., ironic correct sentences), target sentences were syntactically and semantically identical, but differed in their pragmatic meaning. Target sentences consisted of a subject followed by a predicate, which contained a copula verb completed by an adjective, or an intransitive verb followed by an adverbial. In occurrence as predicative completions, adjectives, alike adverbials in general, remain uninflected. For the syntactic condition, a violation of morphosyntactic constraints was induced by inflection of the adjectives and adverbials at the sentence-final position (e.g., *That is richs). Critical information about the utterance meaning (pragmatic manipulation) and sentence correctness (syntactic manipulation) is contained by the target sentence-final word. In the combined condition, ironic sentences consisted of a morphosyntactic anomaly (i.e., ironic incorrect sentences). The experimental design is displayed schematically in Table S2.

Before the experiment, two pretests were carried out on the grammatically correct stimuli in order to control for semantic expectancy, and sentence acceptability of the target sentences. Following Taylor [55], a sentence completion task was employed, in which the items were presented except for the sentence-final word that had to be completed with the most appropriate word. Whenever sentence completions for an ironic and literal sentence were semantically related, sentences were included as experimental items. In this completion task an average cloze probability of 91.8% (SD 8.15) for sentence-final words was obtained. Sentence-final words of ironic sentences were less expected (about 8%) than those of literal sentences (paired t-test on items t(119) = 7.31, p<0.0001). Further, in an acceptability test, in which participants were asked to rate the items on a 5-point-scale (1 for less acceptable, 5 for high acceptable), the degree of sentence acceptability was tested. An average acceptability of 3.7 (SD 0.41) was found, indicating that ironic and literal sentences did not differ in acceptability (paired t-test on items t(119) = 1.22, n.s.).

The 120 experimental sentences were divided into four lists with 60 ironic and 60 literal sentences each, and pseudo-randomized. A target sentence meaning occurred only once per list. All conditions and items were balanced across the lists (i.e., 30 items each). Each participant saw only one list. The four experimental conditions were: literal correct, literal incorrect, ironic correct, ironic incorrect (i.e., combined condition).

The experimental procedure lasted approximately 45 minutes, during which participants were seated in a sound-attenuated cabin facing a computer screen at a distance of about 100 cm. At the beginning of the experiment, participants were instructed to attentively read the stories and to respond to the comprehension task as accurately as possible. The experimental items were presented visually, whereby the context sentences appeared in one block of three to four lines on the screen. Target sentences were presented word by word in a rapid visual presentation mode of 300 ms for words and 200 ms between them. After sentence offset and an inter-stimulus interval of 1500 ms, participants had to respond to the comprehension task with a *yes* or *no* response (maximum response time of 6000 ms). For this task, a test statement describing the prior context had to be judged (for examples, see Table S1). The inter-trial interval was 1000 ms.

### Data Acquisition and Analysis

Behavioral data comprised the judgments on the comprehension task, and were analyzed for each condition separately in a repeated-measure ANOVA.

The electroencephalogram (EEG) was recorded continuously from 52 Ag/AgCl electrodes (i.e., Fp1, Fpz, Fp2, Af7,Af3, Afz, Af4, Af8, F7, F5, F3, Fz, F4, F6, F8, Ft7, Fc5, Fc3, Fcz, Fc4, Fc6, Ft8, T7, C5, C3, Cz, C4, C6, T8, Tp7, Cp5, Cp3, Cpz, Cp4, Cp6, Tp8, P7, P5, P3, Pz, P4, P6, P8, Po7, Po3, Poz, Po4, Po8, O1, Oz, O1 and left mastoid) mounted in an elastic cap (Electro Cap International). To control for eye movements, the bipolar horizontal and vertical electrooculogram was recorded. The sampling rate was 250 Hz, and EEG recordings were referenced to the left mastoid. For the calculation of ERPs, EEG data were averaged for the critical word for each electrode position for each of the four experimental conditions in the time window of −200 ms to 1000 ms relative to the presentation onset of the critical word. Averages included only correctly answered trials that were free from any artifacts (approximately 8% rejections due to ocular or movement artifacts).

**Figure 1:**
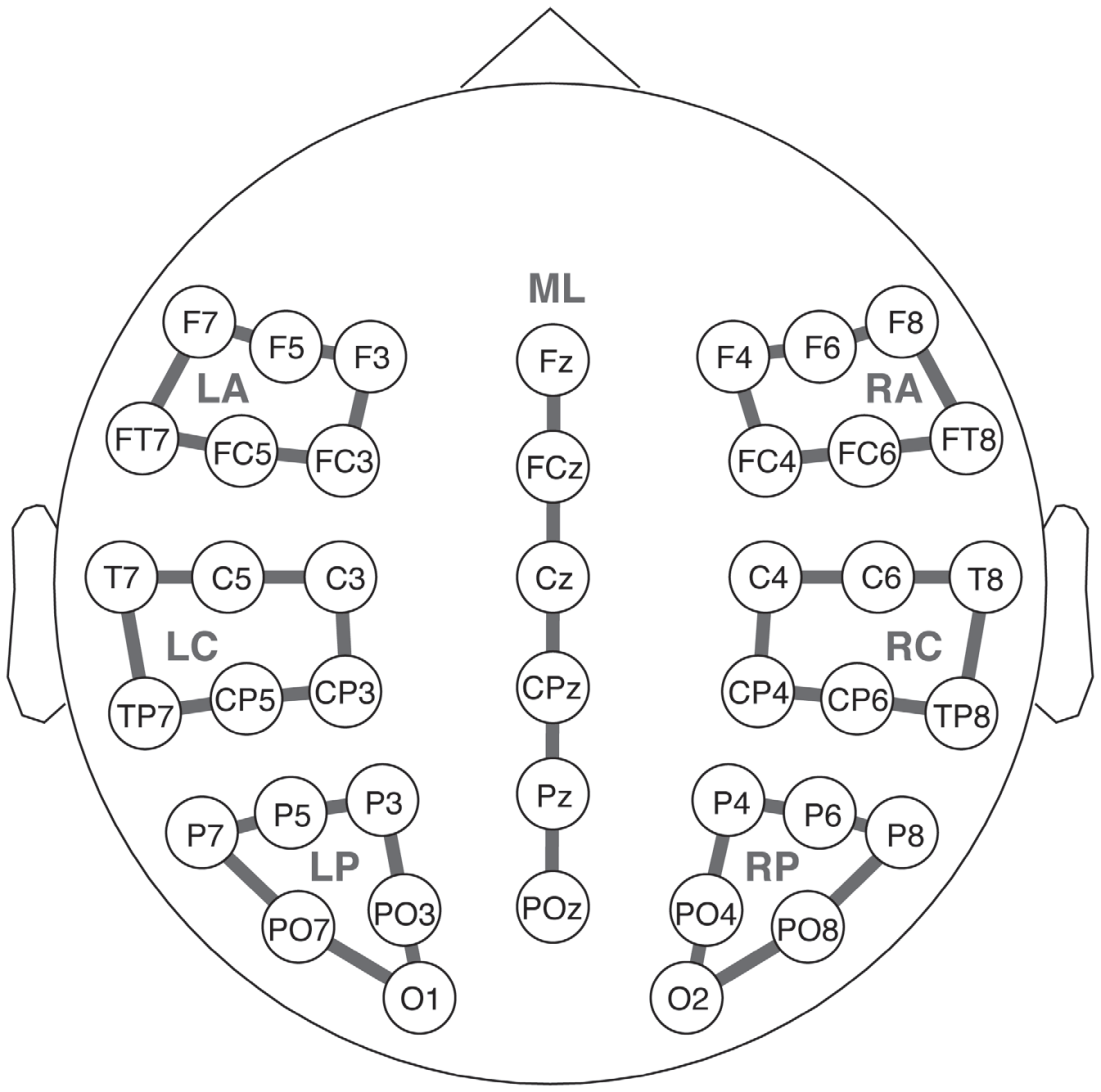
Electrode configuration of the Regions of Interest (ROIs) used for statistical analysis.

For time-frequency analysis of the EEG data, we used the Fieldtrip toolbox for EEG/MEG analysis [56]. Data were filtered with a Hamming-windowed 6^th^-order 0.03-Hz finite-impulse-response high-pass filter to remove any slow drifts. To avoid border artifacts in the power spectra, the experimental trials were cut from the EEG with a large padding (i.e., −4000 ms to 6000 ms). Time-frequency analysis was then performed in 50-ms time steps from −500 ms to 1000 ms, and in 1-Hz steps from 4 Hz to 100 Hz, using Morlet wavelets of seven cycles each [57]. To quantify stimulus-related power changes, power in the stimulus interval (i.e., 0 ms to 1000 ms) was expressed as percentage of change relative to baseline power (i.e., −500 ms to 0 ms). For statistical analysis of potential ERP effects, the following time windows were calculated: *250–400 ms* for the P200 effect in response to the pragmatic condition, *300–500 ms* for the LAN in the syntactic and the combined condition, *500–900 ms* for the P600 in the pragmatic condition, as well as the syntactic and the combined condition. All dependent variables were quantified using multivariate analyses of variance (MANOVAs). The multivariate approach to repeated measurements was used to avoid problems concerning sphericity [58], [59]. For all time windows, an overall MANOVA including all levels of condition (literal correct, ironic correct, literal incorrect, ironic incorrect), as well as planned pairwise comparisons were conducted. For distributional ERP analysis, two topographic factors anterior/posterior (anterior/central/posterior) and hemisphere (left/right) were defined, yielding six different Regions of Interest (ROIs), each containing six electrodes (see Figure 1). Midline electrode positions (i.e., Fz, Fcz, Cz, Cpz, Pz, and Poz) were analyzed separately. Within-subject factors were anterior/posterior (3), hemisphere (2), and condition (4). Whenever the main analysis showed interactions between condition and the topographic factors, further analyses within specific ROIs were carried out. Effects having a significance level of p<0.05, as well as p<0.1 indicating marginally significant effects, are reported. All effects resulting from planned comparisons are corrected by the Bonferroni-Holm procedure.

For statistical analysis of time-frequency data, the relative power change data were averaged across the P600 time window (i.e., 500–900 ms) and frequency band of interest (i.e., theta: 4–7 Hz; alpha: 8–12 Hz). These two frequency bands were chosen based on previous studies reporting power modulations in the respective frequency ranges in response to syntactic and pragmatic information processing (e.g., [36], [48], [50]). In order to capture potential difference in oscillatory power in earlier time windows, the P200 (i.e., 250–400 ms) and the LAN (i.e., 300–500 ms) time window were also analyzed. Because this analysis was aimed to assess differences between the syntax and pragmatic effects (see Results), we first quantified the individual syntax effect by subtracting the literal correct from the literal incorrect condition, and the individual pragmatic effect by subtracting the literal correct from the ironic correct conditions. These two individual difference values for each electrode and frequency band were entered into a cluster permutation paired t-test [60] using a Monte-Carlo simulation with 5000 repetitions to identify significant electrode clusters in each time window and frequency band while controlling for false positives. To this end, the algorithm was set to first identify significant differences at an electrode-level p-value of 0.05, and then search for electrodes that behave similarly at a cluster-level p-value of 0.05. A minimum of two neighboring channels was chosen for cluster detection.

## Results

### Behavioral Data

In the comprehension task, participants performed at ceiling across conditions (mean accuracy rate of 97% (SD 1.83)). Statistical analysis revealed no significant effect of condition (F(3,117) = 1.66, n.s.), which indicates that participants’ performance was comparable across all conditions.

### Electrophysiological Data

**Figure 2:**
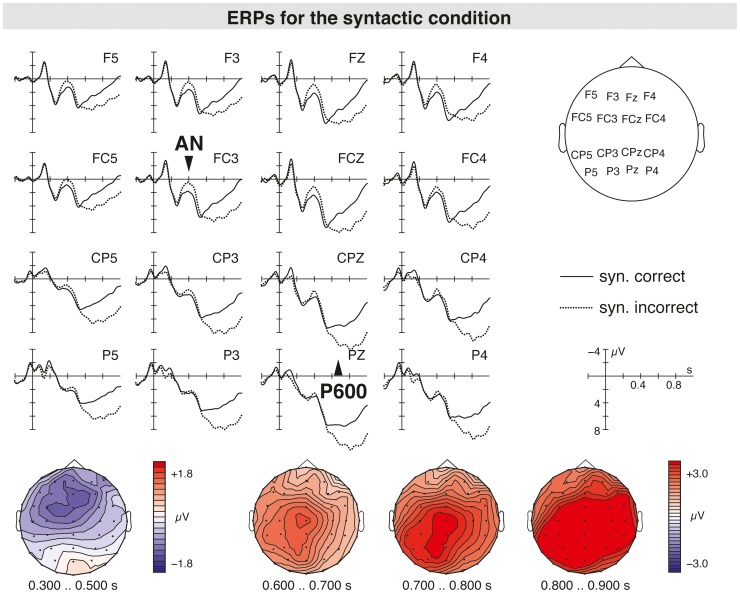
Grand average ERPs elicited by sentence-final words for syntactically correct sentences (solid line) compared to syntactically incorrect sentences (dotted line). The topographic maps of the scalp distribution of the P600 effect are illustrated in the column below.

**Figure 3:**
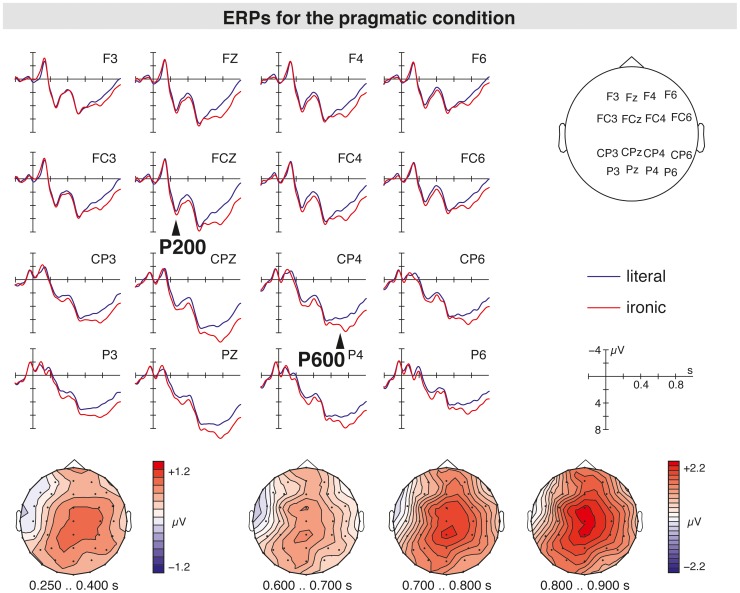
Grand average ERPs elicited by sentence-final words that pointed to a literal (blue line), and an ironic interpretation (red line) with respect to the prior context. The topographic maps in the column below display the scalp distribution of the irony-related P600 component.

**Figure 4:**
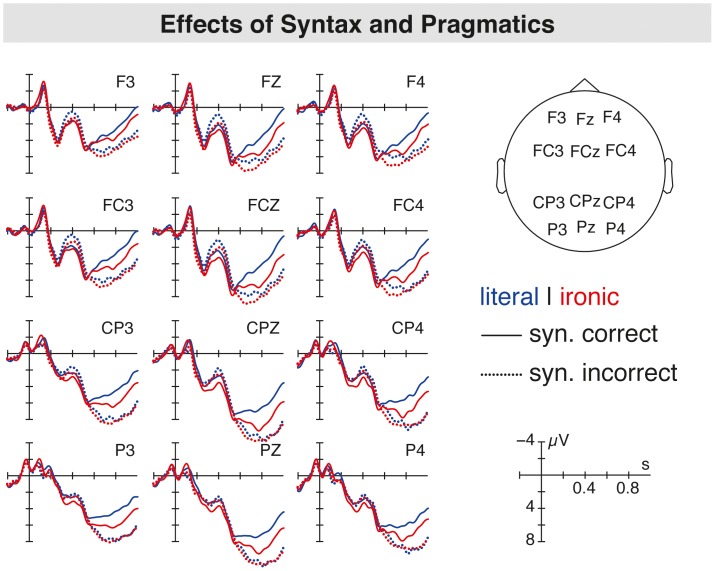
Grand average ERPs analyzed for the critical words of all conditions showing the brain potentials for the syntactic manipulation (correct (solid line) vs. incorrect (dotted line) condition) for both literal (blue line) and ironic (red line) sentences.

The grand average ERPs at the sentence-final word suggested the emergence of a P600 response for both the syntactic anomaly (see Figure 2) and the pragmatic ambiguity (see Figure 3). Most interestingly, when comparing the P600 for the syntactic anomaly (hereafter syntax-related P600) and the P600 for the pragmatic ambiguity (hereafter irony-related P600) there seemed to be topographic differences in the scalp distribution of both positivities. While the syntax-related P600 displayed a widespread scalp distribution, the P600 in response to pragmatically ambiguous sentences was restricted to central and centroparietal electrode positions. When comparing the ERPs for the syntactic with the combined condition (i.e., ironic incorrect sentences), no differences in the P600 amplitudes are seen suggesting the absence of an additive effect (see Figure 4). Moreover, the P600 for both syntactically incorrect literal and ironic sentences was preceded by a LAN peaking around 400 ms in comparison to syntactically correct equivalents. For pragmatically ambiguous sentences, the irony-related P600 seemed to be preceded by a P200 component for irony relative to literal sentences (see Figure 3). An N400 component related to irony was not found.

### The P200∶250–400 ms

The overall statistical analysis of the *250–400 ms* time window revealed a three-way interaction between condition, anterior/posterior, and hemisphere (F(6,34) = 3.34, p<0.01). In the analysis of literal correct to ironic correct sentences, an interaction of condition with anterior/posterior and hemisphere (F(2,38) = 6.20, p<0.005) was found. Follow-up analyses for each ROI separately revealed marginally significant effects of condition in the left parietal (F(1,39) = 2.90, p<0.1) and the right central ROIs (F(1,39) = 3.44, p<0.1) for ironic correct sentences compared to literal correct ones. The analysis of the midline electrodes showed a main effect of condition (F(3,37) = 2.89, p<0.05). In a separate analysis for ironic correct sentences compared to literal correct sentences, an effect of condition was significant (F(1,39) = 4.16, p<0.05), which confirms the presence of a P200 component for irony relative to literal sentences present mainly over central electrode positions.

### The LAN: 300–500 ms

In the time window between *300–500 ms*, the main analysis showed a three-way interaction of condition, anterior/posterior and hemisphere (F(6,34) = 3.78, p<0.01). In the statistical analysis of literal correct versus literal incorrect sentences, a significant interaction of condition with anterior/posterior (F(2,38) = 6.05, p<0.005) was present. This interaction was resolved by anterior/posterior, and revealed significant effects of condition for anterior (F(1,39) = 5.10, p<0.05) and central electrode sites (F(1,39) = 4.25, p<0.05) for literal incorrect sentences compared to equivalent correct sentences. Statistical analysis of the midline electrode positions showed an effect of condition (F(3,37) = 3.61, p<0.05). By analyzing literal incorrect versus correct sentences, a marginally significant effect of condition was found on the midline electrodes (F(1,39) = 3.67, p<0.1). These findings confirm the presence of a bilaterally distributed LAN in response to the syntactic anomaly.

In order to analyze whether the LAN was influenced by pragmatic ambiguity, literal incorrect sentences were compared to ironic incorrect sentences. The statistical analysis revealed no effect of condition, neither on any ROI, nor on the midline electrodes (F(1,39)<2.73, n.s.) suggesting that the LAN did not differ between the syntactic and the combined conditions.

### The P600∶500–900 ms

The main analysis on the *500–900 ms* time window showed an effect of condition (F(3,37) = 14.32, p<0.001), and a significant three-way interaction between condition and the topographic factors anterior/posterior and hemisphere (F(6,34) = 6.78, p<0.001).

### The Syntax-related P600: Literal Syntactic Incorrect versus Correct Sentences

The analysis of literal incorrect compared to literal correct sentences in the 500–900 ms time window showed a main effect of condition (F(1,39) = 19.81, p<0.001) indicating a late positivity effect in response to the syntactic anomaly. No significant interactions of condition with any of the topographic factors were seen (F(2,38) = 1.02–2.45, n.s.), suggesting that the syntax-related positivity had a widespread distribution. Analysis of the midline electrodes also showed a reliable difference of syntactically correct and incorrect sentences (F(1,39) = 22.25, p<0.001), confirming a broad distribution of the syntax-related P600 over anterior and posterior electrode positions alike.

### The Irony-related P600: Ironic Correct versus Literal Correct Sentences

The analysis of ironic correct relative to literal correct sentences showed a significant interaction of condition with anterior/posterior and hemisphere (F(2,38) = 16.57, p<0.001). The resolution of this interaction by the topographic factors showed effects of condition for the right central, and the right and left parietal ROIs (F(1,39) = 4.77–8.61, p<0.05) indicating a P600 for ironic sentences, which was distributed over right central and centroparietal electrode sites. On the midline electrodes, an effect of condition was also present (F(1,39) = 9.57, p<0.005). The analyses indicate that the P600 in response to the pragmatic ambiguity was restricted to central and centroparietal electrode sites.

### Comparing the Scalp Distribution of the Syntax-related and the Irony-related P600: Ironic Correct versus Literal Incorrect Sentences

To test whether the scalp distribution of the syntax-related and the irony-related P600 differed, ERPs for literal incorrect sentences compared to those for ironic correct sentences were analyzed. This analysis showed an effect of condition (F(1,39) = 4.82, p<0.05), and an interaction of condition with anterior/posterior and hemisphere (F(2,38) = 3.50, p<0.05) suggesting that both late positivities differed in their enlargement on the scalp surface.

### Testing for an Additive Effect: Literal Incorrect versus Ironic Incorrect Sentences

In order to test whether the P600 for literal incorrect sentences differed from the P600 for ironic incorrect sentences, these two conditions were analyzed. The statistical analysis revealed neither an effect of condition (F(1,39) = 0.20, n.s.), nor an interaction of condition with any of the topographic factors (F(2,38) = 2.96, n.s.). This implies the absence of an additive effect for the combined condition.

### Comparison of the Syntax and Pragmatics Effects in Time-frequency Space

**Figure 5:**
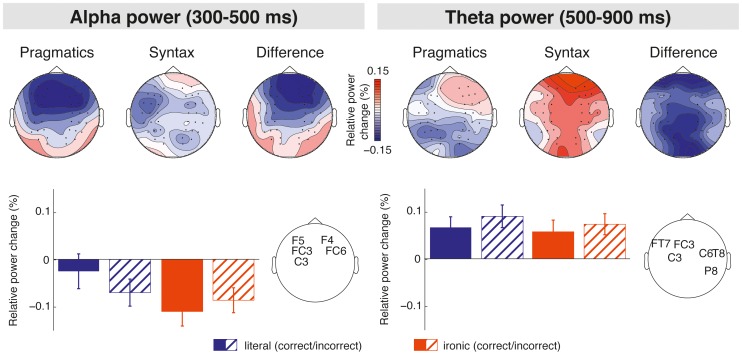
Time-frequency results of the 300–500 ms (left column) and the 500–900 ms (right column) time windows seen for the pragmatics and the syntax effects. The upper panel displays the scalp distribution in the alpha and the theta frequency band, the bottom panel shows the relative power changes at the respective electrode clusters.

The statistical comparison of the syntax (i.e., literal incorrect minus literal correct) and pragmatics (i.e., ironic correct minus literal correct) effects for each time window and frequency band yielded two significant differences between those effects (see Figure 5). For the time window of the LAN (i.e., *300–500 ms*), the syntax and the pragmatics effects were found to significantly differ in the alpha frequency band (t(39) = −16.51, p = 0.05), such that for the syntax effect a less pronounced decrease in alpha power was seen than for the pragmatics effect. For the P600 time window (i.e., *500–900 ms*), the syntax and the pragmatics effects were found to significantly differ in the theta frequency band (t(39) = −14.58, p<0.05), such that the syntax effect revealed a stronger increase in theta power as compared to the pragmatics effect. For the P200 time window no effects reached significance.

## Discussion

The current study investigated late positivities evoked by syntactic anomaly and pragmatic ambiguity in order to scrutinize the electrophysiological and functional characteristics of the P600 related to language comprehension. The question whether the P600 is a reflection of common neurocognitive processes, or whether this ERP component comprises distinct brain responses sensitive to particular types of information, was addressed. In addition, the neural oscillatory activity in the P600 time window was analyzed to explore whether the synchronization and desynchronization patterns differ between syntactic and pragmatic information processing. ERPs in response to the syntactic anomaly (i.e., morphosyntactic violation) revealed a so-called syntax-related P600 component that was preceded by a LAN. ERPs in response to the pragmatic ambiguity (i.e., irony) showed a P200 component followed by a so-called irony-related P600 component. For both P600 effects, a similar onset latency of around 500 ms post-stimulus was observed. Most interestingly, in a direct comparison of the syntax-related and the irony-related P600, distributional differences were obtained, showing a widespread scalp distribution of the syntax-related P600, but a more restricted distribution of the irony-related P600, constrained to central and centroparietal electrode sites. In prior research, both P600 topographies most robustly occurred for the processing of morphosyntactic violations (e.g., [12], [61]) as well as visually presented pragmatic ambiguities (see [33], [34]). The observed ERP data, hence, may support an involvement of different neural networks in the emergence of the syntax-related and the irony-related P600. In support of this interpretation, the current oscillatory data indicated a different pattern of neural oscillatory activity in the theta frequency band within the P600 time window in response to syntactic and pragmatic information. For the syntax effect (i.e., literal incorrect versus literal correct sentences), a stronger increase in theta power was obtained, as opposed to the pragmatics effect (i.e., ironic correct versus literal correct sentences). In addition, differences in the neural oscillations were also found in the LAN time window, showing a lower decrease of alpha power for the syntax effect in comparison to the pragmatics effect. This finding suggests stronger desynchronization in response to the pragmatic ambiguity than the syntactic anomaly. In view of later processing mechanisms reflected by P600, the observed differences in theta activity imply different processes of synchronization and desynchronization of the underlying neural assemblies, potentially speaking for an involvement of different neural networks in the processing of syntactic and pragmatic information. An amplitude increase in the theta band has already previously been reported for syntactic violations [46]. Changes in theta power were also reported for semantic [49], [62] and pragmatic anomalies [36] suggesting a strong relation of this frequency band to language comprehension. The obtained differences in theta power seen for syntactic and pragmatic information still imply that there seemed to be no unified processing mechanism supporting both structural repair and ambiguity resolution. The observation of distributional differences between the syntax-related and irony-related P600 in combination with differences in the neural oscillatory activity strongly suggest an engagement of different neurocognitive processes in the comprehension of different types of linguistic information. With regard to previous ERP findings, the present results accord with accounts of rather specific processing mechanisms engaged during later phases of language comprehension [7], [63]. In a previous study examining P600 responses for syntactically anomalous and syntactically complex sentences, Friederici et al. [14] reported distinct P600 effects related to syntactic repair and syntactic integration. Similarly to the current data, both P600 effects differed in their scalp distribution, suggesting that different neural structures underlie the processing of both types of syntactic information. In addition, in a recent study by Gouvea et al. [6], P600 effects for three different types of syntactic information were compared, that is, syntactic violations, garden path sentences, and long-distance dependencies. The P600 elicited by long-distance dependencies varied in scalp distribution and amplitude from the P600 evoked by ungrammatical and garden path sentences showing that the P600 is affected by the diverse underlying syntactic processes. In a recent review on ‘semantic P600’ effects, Brouwer et al. [21] propose that the P600 reflects an updating of the mental representation, whereby its electrophysiological characteristics might vary due to specific subprocesses required for constructing coherent sentence representations. This interpretation of P600 effects is supported by the current findings implying that the P600 seems to reflect functionally distinct processes related to language comprehension. Besides, the observation of earlier differences in the ERPs (i.e., P200 and LAN) and the oscillatory data (i.e., changes in alpha power) imply an involvement of distinct processing mechanisms present already during earlier phases of syntax and pragmatics processing. The following section discusses the implications for the assumed neurocognitive processes associated with the comprehension of syntactic and pragmatic information.

### The Processing of Syntactic Information

For the morphosyntactic anomaly, a biphasic ERP pattern consisting of a LAN and a subsequent P600 effect was obtained. This finding is in accordance with ERP findings seen for violations of morphosyntactic constraints [13], [64]. A comparable bilateral LAN response has previously been reported for morphosyntactic anomalies, in particular for agreement violations of number and gender [65], [66], as well as for word category violations [12]. Such LAN effects have been associated with syntactic analysis processes in diagnosing morphosyntactic errors, as well as other types of syntactic errors [53]. The morphosyntactic anomaly applied in the current study included a violation of morphosyntactic constraints: The target sentence-final words, which are expected to remain uninflected, were inflected by addition of an inflectional affix to the word stem, resulting in ungrammatical sentence completions. Consistent with the afore-mentioned studies, the obtained LAN seems to be associated with morphosyntactic analysis of the constraint violation present at the critical word.

In addition, the P600 component following the LAN showed a close resemblance to syntax-related P600 effects according to its latency, amplitude and sensitivity. The current P600, hence, might index a reanalysis of the anomalous sentence structure in order to establish a syntactically coherent sentence representation [17]. This observed late positivity showed a large amplitude, comparable to P600 effects seen for number violations in sentences such as *“The elected officials hopes to succeed”* [51]. In the present study, the violation of morphosyntactic constraints possibly set up an outright syntactic violation, since in German, predicative complements and adverbs, as employed for the target sentence endings, remain uninflected according to grammatical constraints. Such type of morphosyntactic anomaly might have profoundly disrupted sentence processing, resulting in large amplitude of P600.

### The Processing of Pragmatic Information

ERPs seen for the pragmatic ambiguity revealed an enhanced P200 and a subsequent P600 for ironic relative to literal sentences, in the absence of an irony-related N400 component. The finding of an early ERP response (i.e., P200 component) emerging around 250 ms post-stimulus onset suggests that the comprehension of literal and ironic language already differed during earlier phases of processing. For both literal and ironic sentences strong contextual support was provided, so that on the basis of this information, a semantic-pragmatic prediction for respective utterance meanings might have been occurred. The observed P200 effect elicited by the pragmatic ambiguity might indicate semantic association processes when encountering a critical word biasing a non-literal utterance interpretation. A sensitivity of P200 to aspects of semantic information processing at the sentence level has been substantiated in visual field studies [67], [68]. Therein, P200 effects have been observed for words completing strongly constrained sentence contexts compared to weakly constrained ones, which have been associated with the apprehension of an upcoming stimulus [68]. In contrast to the reported P200 effects, the present finding of a P200 was not related to differences in contextual constraints, but to content-related variations of contextual information indicating an early effect of context when encountering a pragmatic ambiguity.

The irony-related P600 following the P200 might be a reflection of pragmatic reanalysis processes, allowing for a coherent utterance interpretation. In line with recent ERP studies, comparable irony-related P600 effects were robustly observed in the absence of an N400 effect [33], [34], [36]. When encountering the critical information for non-literal sentence interpretations, the processing of lexical-semantic information apparently seems to be not more difficult than for literal language. The emergence of an early effect of context (i.e., P200 component) implies that the processing system seems to distinguish between potential ironic and literal utterance meanings as early as around 250 ms after stimulus onset. On the basis of contextual information, semantic associations might have occurred, which could have facilitated later lexical-semantic processes. For successful comprehension of the intended utterance meaning, however, a reanalysis of the literal sentence meaning seems to be required. In such reanalysis, pragmatic knowledge and contextual information might have been taken into account, enabling an utterance interpretation on the message-level and a derivation of a speaker’s communicative intent [69]. The observation of an irony-related P600 might be associated with such processes of reanalysis of pragmatically ambiguous sentences in order to establish adequate utterance meanings for this sentence context. This interpretation is in line with the suggestion that the irony-related P600 may be associated with the integration of linguistic and contextual information [36].

Comparing the finding of an irony-related P600, to studies reporting ‘semantic P600’ effects in absence of any semantic or syntactic anomaly shows that this positive brain potential reliably emerges for non-literal language and pragmatically complex phenomena [35], [37], [70]. ‘Semantic P600s’ were also seen in response to thematic violations of syntactically well-formed sentences, which have been interpreted as a function of structural processes in assigning appropriate thematic roles (e.g., [24], [25], [40]). Such an account, however, cannot explain P600 effects in response to semantically and syntactically non-anomalous sentences, such as figurative language (e.g., [34], or jokes [37]. The P600 in response to irony is unlikely a reflection of structural processes, since pragmatic ambiguity of the intended utterance meaning, in absence of thematic role violations, cannot be resolved by restructuring the sentence.

## Conclusion

The present study examined processing mechanisms reflected in the P600 component seen in response to the processing of syntactic and pragmatic information. Both the ERP and neural oscillatory data imply that at least partially different neural assemblies contributed to the syntax and pragmatics effects suggesting an engagement of different neurocognitive processes in the processing of syntactic and pragmatic information. During later phases of processing, the comprehension of syntactic information might engage processes of structural reanalysis in establishing a coherent sentence structure (see e.g., [17]), whereas the comprehension of pragmatic ambiguities seems to be associated with pragmatic reanalysis allowing an adequate utterance interpretation [33], [34].

## Supporting Information

Table S1 Examples of ironic (pragmatically ambiguous) stimuli (a) and literal stimuli (b) including examples of the experimental test statements.
(DOC)

Table S2 Experimental design with the factors syntax and pragmatics as used in the current study.
(DOC)
