# Supplementary material for: Distinguishing Neurocognitive Processes Reflected by P600 Effects: Evidence from ERPs and Neural Oscillations
Source: PLoS One. 2014 May 20;9(5):e96840. doi: 10.1371/journal.pone.0096840 (PMC4028180; doi:10.1371/journal.pone.0096840)
Supplement: Table S1 — Examples of ironic (pragmatically ambiguous) stimuli (a) and literal stimuli (b) including examples of the experimental test statements. (DOC) [file pone.0096840.s001.doc]

**Table S1**. Examples of ironic (pragmatically ambiguous) stimuli (a) and literal stimuli (b) including examples of the experimental test statements.

|  | **Contextual information** | **Target sentence** (correct/incorrect) | **Test statement** |
| --- | --- | --- | --- |
| 1a | Kristina und Björn fahren zusammen an die Ostsee, wo sie sich eine kleine Frühstückspension nehmen. Am Morgen erhalten beide zwei Toasts mit etwas Marmelade. Björn sieht erstaunt auf die winzige Portion und sagt: | Das ist hier ja sehr reichlich/  *reichlichen | Es gibt nur ein kleines Frühstück. |
| 2a | Annelie und Simone wollen zusammen kochen. Annelie soll die Kartoffeln schälen. Das tut sie auch sehr sorgfältig, aber redet die meiste Zeit über. Als Simone wieder zu ihr hinsieht, hat sie erst zwei geschält. Entgeistert meint sie: | Du bist ja schon fertig/  *fertigen | Simone wartet auf die Kartoffeln. |
| 3a | Auf der Tagung hält Professor Schmidt eine komplizierte Rede. Er macht viele Gedankensprünge, so dass man nur schwerlich folgen kann. Ein Teilnehmer hat es schon längst aufgegeben und murmelt am Ende verärgert: | Das war wirklich sehr aufschlussreich/  *aufschlussreichen | Der Vortrag war unstruktiert. |
| 1b | Björn möchte Kristina gern zum Essen einladen. Im Restaurant haben sie sich schnell für die Lasagne entschieden und genießen den Rotwein. Als das Essen kommt, sieht Kristina erschrocken auf die große Portion und sagt: | Das ist hier ja sehr reichlich/  *reichlichen | Kristina bekam ein große Portion. |
| 2b | Die kleine Amelie hat ihre Aufgaben immer schnell erledigt. Selbst beim Basteln hat sie den komplizierten Kranich rasch zusammengefaltet. Die Betreuerin sieht auf den Vogel und sagt überrascht: | Du bist ja schon fertig/  *fertigen | Amelie ist meist schnell fertig. |
| 3b | Annelie und Kristina hören einen Vortrag über die Veränderung des Klimas. Interessiert folgen sie den detaillierten Ausführungen des Referenten. Am Ende sagt Annelie beeindruckt: | Das war wirklich sehr aufschlussreich/  *aufschlussreichen | Annelie fand den Vortrag interessant. |
| APPROXIMATE TRANSLATION OF THE STIMULI FROM GERMAN | | | |
| 1a | Christina and Bjorn are on holiday at the sea, where they are staying in a small hotel. In the morning they receive only toast with jam for breakfast. Bjorn looks quite disappointed at his serving and says: | That is rich/  *richs | They had only a small breakfast. |
| 2a | Ann and Simona decided to cook dinner. Ann offered to peel the potatoes but was taking and talking. Simona, quite hungry by now, looked at her and saw only two peeled potatoes. Grumpily she says: | You are so quick/  *quicks | Simona is waiting for the potatoes. |
| 3a | In the introductory seminar Professor Taylor gave a very complicated talk. He made many mental leaps, so that most of the students could not follow. One of the listener had already given up, and, annoyed, murmured: | This is so informative/  *informatives | The talk was unstructured. |
| 1b | Bjorn invites Christina to a new restaurant, where they have quickly ordered their dishes, and are now enjoying a glass of red wine. When the dishes arrive, Christina looks surprised at her large serving and says: | That is rich/  *richs | Christina had a large serving. |
| 2b | Little Ann is always very quick doing her homework. Even when she does handicrafts, she can very quickly fold a paper crane. As her aunt watched her folding the bird, she is astonished and says: | You are so quick/  *quicks | Amelie has often quickly finished. |
| 3b | At the Technology Institute Annelie listened to a talk about the latest developments. Interested in the topic, she followed all the statements. She found out many new things and, impressed, said: | This is so informative/  *informatives | Annelie was impressed by the talk. |
