# Supplementary material for: Distinguishing Neurocognitive Processes Reflected by P600 Effects: Evidence from ERPs and Neural Oscillations
Source: PLoS One. 2014 May 20;9(5):e96840. doi: 10.1371/journal.pone.0096840 (PMC4028180; doi:10.1371/journal.pone.0096840)
Supplement: Table S2 — Experimental design with the factors syntax and pragmatics as used in the current study. (DOC) [file pone.0096840.s002.doc]

**Table S2**. Experimental design with the factors syntax and pragmatics as used in the current study.

|  | **Syntax** | |
| --- | --- | --- |
| **Pragmatics** | syntactically correct | syntactically incorrect |
| literal | *literal correct* | *literal incorrect* |
| ironic | *ironic correct* | *ironic incorrect* |
